# Exercise to Reduce Mobility Disability and Prevent Falls After Fall-Related Leg or Pelvic Fracture: RESTORE Randomized Controlled Trial

**DOI:** 10.1007/s11606-020-05666-9

**Published:** 2020-02-03

**Authors:** Catherine Sherrington, Nicola Fairhall, Catherine Kirkham, Lindy Clemson, Anne Tiedemann, Constance Vogler, Jacqueline C. T. Close, Sandra O’Rourke, Anne M. Moseley, Ian D. Cameron, Jenson C. S. Mak, Stephen R. Lord

**Affiliations:** 1 Institute for Musculoskeletal Health, School of Public Health, University of Sydney, Sydney, NSW Australia; 2 Faculty of Health Sciences, University of Sydney, Sydney, NSW Australia; 3 Northern Clinical School, Sydney Medical School, The University of Sydney, Sydney, NSW Australia; 4 Department of Aged Care, Royal North Shore Hospital, St Leonards, NSW Australia; 5 Prince of Wales Clinical School, University of New South Wales, Randwick, NSW Australia; 6 Neuroscience Research Australia, University of New South Wales, Randwick, NSW Australia; 7 John Walsh Centre for Rehabilitation Research, Kolling Institute, Faculty of Medicine and Health, The University of Sydney, St Leonards, NSW Australia; 8 Rehabilitation and Aged Care Services, Gosford Hospital, Gosford, NSW Australia

**Keywords:** randomized controlled trial, exercise, hip fracture, fall prevention

## Abstract

**Background:**

Disability and falls are common following fall-related lower limb and pelvic fractures.

**Objective:**

To evaluate the impact of an exercise self-management intervention on mobility-related disability and falls after lower limb or pelvic fracture.

**Design:**

Randomized controlled trial.

**Participants:**

Three hundred thirty-six community dwellers aged 60+ years within 2 years of lower limb or pelvic fracture recruited from hospitals and community advertising.

**Interventions:**

RESTORE (Recovery Exercises and STepping On afteR fracturE) intervention (individualized, physiotherapist-prescribed home program of weight-bearing balance and strength exercises, fall prevention advice) versus usual care.

**Main Measures:**

Primary outcomes were mobility-related disability and rate of falls.

**Key Results:**

Primary outcomes were available for 80% of randomized participants. There were no significant between-group differences in mobility-related disability at 12 months measured by (a) Short Physical Performance Battery (continuous version, baseline-adjusted between-group difference 0.08, 95% CI − 0.01 to 0.17, *p* = 0.08, *n* = 273); (b) Activity Measure Post Acute Care score (0.18, 95% CI − 2.89 to 3.26, *p* = 0.91, *n* = 270); (c) Late Life Disability Instrument (1.37, 95% CI − 2.56 to 5.32, *p* = 0.49, *n* = 273); or in rate of falls over the 12-month study period (incidence rate ratio 0.96, 95% CI 0.69 to 1.34, *n* = 336, *p* = 0.83). Between-group differences favoring the intervention group were evident in some secondary outcomes: balance and mobility, fall risk (Physiological Profile Assessment tool), physical activity, mood, health and community outings, but these should be interpreted with caution due to risk of chance findings from multiple analyses.

**Conclusions:**

No statistically significant intervention impacts on mobility-related disability and falls were detected, but benefits were seen for secondary measures of balance and mobility, fall risk, physical activity, mood, health, and community outings.

**Trial Registration:**

Australian New Zealand Clinical Trials Registry: ACTRN12610000805077

**Electronic supplementary material:**

The online version of this article (10.1007/s11606-020-05666-9) contains supplementary material, which is available to authorized users.

## INTRODUCTION

Fall-related lower limb and pelvic fractures impact on individuals, their carers, health services, and the community.^1–3^ Up to 60% of hip fracture survivors do not regain pre-fracture levels of activity or mobility and are at high risk of further falls.^3^ Costs associated with fall-related fractures are increasing rapidly along with the proportion of older people in the global population.^4^

Outcomes after fall-related fractures can be improved with well-designed exercise-based intervention programs^1, 5–7^ but the optimal approach is not yet clear.^8^ There are strong effects on mobility and function from intensive supervised center-based exercise interventions,^9, 10^ but effectiveness of more economically viable home-based programs is inconsistent.^8, 11, 12^ One high-quality trial found a 6-month home exercise program, taught by a physiotherapist and undertaken with minimal supervision, improved mobility in older people after hip fracture.^13^ This built on previous trials in which home exercise programs taught by physiotherapists improved short-term mobility outcomes after hip fracture.^14, 15^ Motivational interviewing^16^ and other self-management approaches may enhance uptake of home exercise after hip fracture^17^ but are yet to be evaluated in large-scale trials.

Few trials have evaluated the effect of exercise programs on the prevention of further falls in fracture survivors.^11, 18^ One trial found prescription of home-based exercise by physiotherapists prior to hospital discharge was feasible and reduced falls in the following 12 months.^18^ We found that a home exercise program, that did not specifically include fall prevention advice, improved mobility but increased falls in older people who had recently returned home after hospital stays.^19^ In the general older community, falls can be prevented with exercise targeting balance and functional activities.^20, 21^ The *Stepping On* self-management-focused group-based program can prevent falls and improve self-efficacy in older people with past falls.^22^

Our multi-disciplinary team of investigators designed the Recovery Exercises and STepping On afteR fracturE (RESTORE) home-based intervention program, which includes exercise and fall prevention education and aims to reduce mobility-related disability and falls. A self-management approach aims to enhance uptake of the exercise program using (optional) goal-setting and the education aspect involves *Stepping On* program attendance or individualized information on safe community mobility and other risk factors for falls.

### Objectives

The primary objective of this study was to estimate the average effect of the RESTORE program compared with usual care on mobility-related disability and rate of falls in older people aged 60 years or more with a recent fall-related lower limb or pelvic fracture.

Secondary objectives were to: estimate the average effect of the RESTORE program compared with usual care on balance and mobility, falls (proportion of fallers, type of fall, consequences of falls), fall risk (Physiological Profile Assessment tool), physical activity, walking aid use, pain, body mass index, fall-related self-efficacy, mood, self-rated health, stage of motivational readiness for change, and community participation in people aged 60+ with a recent fall-related lower limb or pelvic fracture, and to describe the safety and acceptability of the RESTORE program.

## METHODS AND DESIGN

### Trial Design

A parallel-group randomized controlled trial with equal allocation to intervention and control groups was conducted in people aged 60+ years with fall-related lower limb or pelvic fracture in the 2 years before recruitment. Ethical approval was obtained from the Northern Sydney Central Coast Human Research Ethics Committee (0905-089M) and local governance committees at the recruitment sites prior to data collection. Study recruitment was undertaken between April 2010 and November 2014. Follow-up was completed in December 2015. The study was registered in the Australian New Zealand Clinical Trials Registry (ACTRN12610000805077). The study protocol is published.^23^

### Participants

Participants were recruited from 11 hospitals in New South Wales, Australia. Potential participants were identified in hospital via discussion with hospital staff and review of ward lists. Permission for study staff to contact potential participants after return home was sought while they were in hospital. Letters were also sent to potentially eligible people identified from hospital databases at several of the sites. Advertisements for study participants were placed on notice boards in hospitals, community centers, and in local newspapers.

People were not eligible to participate in the study if they resided in a high-care residential facility (nursing home); had a cognitive impairment (a Mini-Mental State Examination (MMSE) score of less than 24); had insufficient English language to understand study procedures; were unable to walk more than 10 m despite assistance from a walking aid and/or another person; had a medical condition precluding exercise (e.g., unstable cardiac disease or progressive neurological disease); or were currently receiving a treatment program from a rehabilitation facility.

### Interventions

The intervention program is detailed in Supplementary Table 1. Two experienced physiotherapists visited participants’ homes, up to ten times in the 12-month study period, to prescribe and modify a home exercise program. Participants were asked to undertake a 20- to 30-min program of lower limb balance and strengthening exercises at least three times per week at home for 12 months. The exercises were primarily conducted while standing and were based on the Weight-Bearing Exercise for Better Balance program, available at www.webb.org.au. The Physical Activity Stage of Change model^24^ guided the physiotherapists’ approach to encouraging ongoing home-based exercise participation.

Participants also received advice about fall prevention based on the *Stepping On* program. Where possible, participants attended a group-based program (seven 2-h group sessions) delivered as part of the state-wide program roll-out by the New South Wales Health Department. If a participant did not wish to attend the *Stepping On* program, a suitable group was not available or transport to the group was too difficult, individualized fall prevention advice was given by the physiotherapist in the home visit sessions. This advice focused on safe mobility and also covered safe footwear, maximizing vision to reduce fall risk and safe medication use.

Participants in both groups received an education booklet about fall prevention and all usual care from health and community services.

### Outcomes

Data were collected from medical records, postal questionnaires and calendars, interviews (by phone and in person), and physical assessments. Information on medical history, diagnoses, and medications were collected from medical records while participants were in hospital or from hospital discharge summaries. Baseline assessments were conducted in participants’ homes, prior to randomization and assessed demographic information and outcomes. Follow-up assessments were conducted 12 months after randomization by physiotherapists and trained research assistants who were blinded to group allocation. Participants were given calendars and questionnaires (with questions about difficulty performing mobility tasks) at the time of the baseline assessment and asked to record falls on the calendars and answer the questionnaire each month and return in pre-paid envelopes to the research center. Participants who did not return calendars or questionnaires were telephoned for the information. Participants who reported falling were telephoned to seek more information about the circumstances and consequences of the fall.

The primary outcome measures were *mobility-related disability* and the *rate of falls* over the 12-month study period. *Mobility-related disability* was assessed at 12-month post-randomization with three measures: (a) the performance-based Short Physical Performance Battery (SPPB); (b) the self-reported Activity Measure Post Acute Care (AM-PAC); (c) the self-reported Late Life Disability Instrument (LLDI). Using the World Health Organization’s International Classification of Functioning Disability and Health (ICF) terminology, these were measures of mobility performance, activity limitation, and participation restriction respectively.

The SPPB was measured at baseline and at 12 months after randomization. It involves the timed performance of three mobility tasks: the ability to stand for up to 10 s with feet in three positions (together side by side, semi-tandem and tandem), 4-m walk, and time to rise from a chair five times. The primary analysis was conducted using the continuously-scored version of this tool (also referred to as the lower-extremity Continuous Summary Performance Score, which uses the time taken to complete mobility tasks. Scores range from 0 (worst performance) to 3 (best performance) and are made up of a scaled score for each task.^25^

The AM-PAC assesses difficulty with the performance of daily tasks. The computerized adaptive testing version of the AM-PAC was administered by phone at baseline, 3, 6, 9, and 12 months. This software chooses questions from an item bank on the basis of participant responses to previous questions to ensure questions asked are appropriate to the person’s level of physical functioning. The sum of the “basic mobility” and “daily activity” components was used as a primary outcome.

The Late Life Disability Instrument (LLDI), assessed at baseline and 12 months, evaluates self-reported limitations (capability) and frequency (performance) of participating in 16 major life tasks, and roles each measured on a five-point scale. The frequency and limitation dimensions are each transformed to a 0 to 100 scale, with higher scores indicating higher levels of participation.

The *rate of falls* was measured using monthly calendars. A fall was defined as an incident in which the body unintentionally came to rest on the ground or other lower level which was not as a result of a violent blow, loss of consciousness or sudden onset of paralysis as in a stroke or an epileptic seizure.^26^

Secondary outcome measures were additional measures of balance and mobility (performance, self-reported activity ease, self-reported participation), additional measures of falls, fall risk, physical activity^28^, pain, body mass index, fall-related self-efficacy, mood, self-rated health, stage of motivational readiness for change^29^ and community outings. These measures aimed to enhance understanding of the effects of the exercise program on multiple factors that contribute to functioning and quality of life. Please see Supplementary Table 2 for information about the secondary outcome measures. Frailty, health service use, quality of life outcomes, and an economic evaluation will be reported separately.

Participants were advised to telephone study staff if they experienced any adverse effects that may have resulted from the exercise program, such as chest pain, or musculoskeletal soreness lasting for more than 48 h and interfering with daily activities or requiring medical attention.

### Sample Size

The study was powered on the basis of the primary *rate of falls* outcome. Sample size calculations indicated that 350 participants (175 per group) would be required for 80% power to detect as significant at the 5% level a 30% reduction in the rate of falling (i.e., an incidence rate ratio (IRR) of 0.70 using negative binomial regression analysis) in the 12-month follow-up period. This number would provide 90% power to detect a statistically significant between-group difference of 10% in SPPB (continuous version). For these calculations, we assumed an α of 0.05, non-compliance of 15% and a drop-out rate of 15%.

### Randomization and Blinding

After consent and completion of the baseline assessment, participants were formally entered into the study and randomized to intervention or control groups. Randomization order was determined using a computer-generated random number schedule with randomly permuted block sizes of 2–6. Allocation was concealed by using central randomization performed by an investigator (CS) not involved in assessments or recruitment. Treatment allocation tables were inaccessible to recruitment staff. Study staff who conducted interviews and assessments, received calendars and questionnaires, made phone calls and entered data were unaware of group allocation. Participants were instructed not to inform the assessors of their intervention status, and all exercise equipment was removed prior to the final assessment. Data analysis for the primary outcomes was undertaken blinded to group allocation.

### Statistical Methods

Analyses were conducted according to the pre-defined statistical analysis plan (see supplementary file) on an intention-to-treat basis (using all available data and analyzing participants in the groups to which they were originally assigned) and were unadjusted except where indicated. For all variables, between-group differences and 95% confidence intervals were calculated. The effect of group allocation on continuous outcomes was estimated using general linear models with pre-test performance as a covariate (ANCOVA). The number of falls per person-year was analyzed using negative binomial regression to estimate the between-group difference in fall rate. Exploratory analyses investigated the rate and proportion of people experiencing indoor falls, outdoor falls, falls requiring medical intervention (i.e., local doctor visit, emergency department visit, hospital admission), falls requiring hospital admission, and falls resulting in fractures as well as the impact of controlling for exposure to physical activity (a) overall self-reported activity and (b) planned self-reported activities multiplied by days of falls follow-up as an exposure term in the negative binomial regression models). For the dichotomized secondary outcomes, log binomial regression models were used to compare the proportion of participants in each group, with baseline score as a covariate. Ordinal logistic regression^27^ was used to compare the groups for ordinal outcomes, with baseline score as a covariate and testing of the “proportionality” assumption using the likelihood ratio test. Pre-planned sub-group analyses used interaction terms in the models to assess whether there was a differential effect of the intervention on the primary outcomes on the basis of number of past falls (0–1 versus 2 or more), cognitive impairment (1 or more adjusted errors on the Short Portable Mental Status Questionnaire (SPMSQ) versus no errors), and baseline gait speed (as a continuous interaction term and dichotomized above and below the median). Data were coded to permit blinding to group allocation in the statistical analysis of the primary outcomes. Analyses were conducted using the Stata software package, College Station, TX.

## RESULTS

### Participant Recruitment and Flow

**Figure 1:**
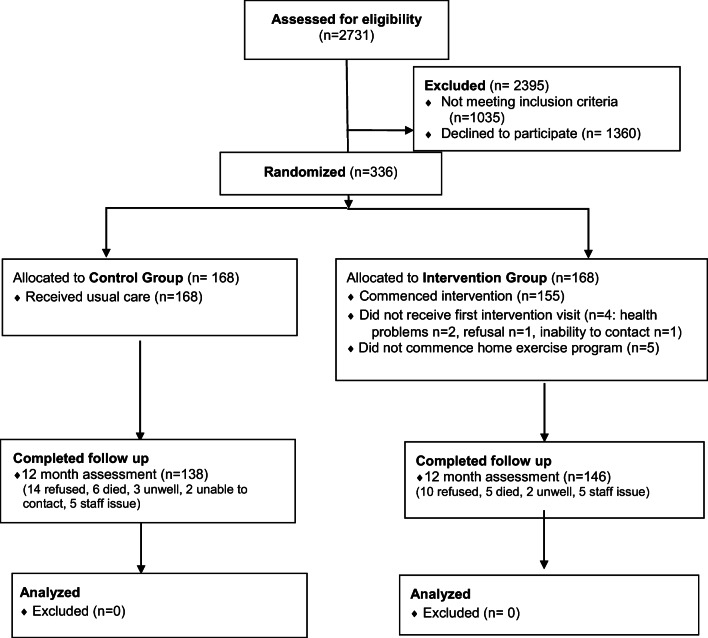
Overview of the flow of participants.

Recruitment was ceased 14 participants short of the original target due to exhaustion of study funds. Supplementary Figure 1 overviews study recruitment methods. Figure 1 shows the flow of participants through the study. Twelve-month assessment was completed by 284 participants (85% of those randomized) and data for all primary outcomes were available for 270 people (80% of those randomized).]-->

### Baseline Data

The average age of the 336 study participants was 78 years (SD 9, range 59–99) and 254 (76%) were women. Table 1 provides a summary of the baseline demographics. Baseline scores for all outcomes are shown in Tables 2 and 3. There were no important differences between the groups at baseline.

**Table 1:** Baseline Characteristics of Participants (*n* = 336)

Characteristic	Control (*n* = 168)	Intervention (*n* = 168)
Age, years, mean (SD)	77.8 (8.6)	77.6 (8.9)
Time since fracture, days, mean (SD)	397.0 (223.5)	407.9 (210.0)
< 3 months, *n* (%)	13 (8)	11 (7)
4–6 months, *n* (%)	31 (18)	24 (14)
7–12 months, *n* (%)	49 (29)	47 (28)
13–18 months, *n* (%)	35 (21)	49 (29)
19–24 months, *n* (%)	40 (24)	37 (22)
Cognition, SPMSQ^a^ adjusted errors, mean (SD)	0.48 (0.85)	0.41 (0.88)
Females, *n* (%)	129 (77%)	125 (74%)
One or more falls in previous 12 months, *n* (%)	117 (69%)	123 (72%)
Medications, number, mean (SD)	6.5 (4.1)	6.5 (3.7)
Living in low-care residential facility (hostel), *n* (%)	8 (5%)	6 (4%)
Co-morbidities^b^, number, mean (SD)	8.2 (3.3)	7.9 (3.5)
Fracture leading to study eligibility, *n* (%)		
Hip	97 (57%)	97 (57%)
Pelvis	16 (10%)	15 (9%)
Knee	10 (6%)	11 (7%)
Tibia/fibula	16 (10%)	16 (10%)
Ankle	19 (11%)	22 (13%)
Heel/foot bones	7 (4%)	4 (2%)
Sacrum	3 (2%)	3 (2%)
Height, cm, mean (SD)	162.2 (9.8)	161.8 (9.2)
Weight, kg, mean (SD)	69.7 (18.8)	68.2 (16.7)

^a^Short Portable Mental Status Questionnaire, errors adjusted for education

^b^Total co-morbidities from a list of 28 conditions

**Table 2:** Baseline and 12-Month Scores on Primary Outcomes of Mobility-Related Disability

	Intervention (*n* = 168), mean (SD), *n* or *n* (%)		Control (*n* = 168), mean (SD), *n* or *n* (%)		12-month between-group difference adjusted for baseline (95% CI), *p*, *n*
	Baseline *n* = 168	12 month *n* = 146	Baseline *n* = 168	12 month *n* = 138	
Performance of mobility tasks ^a^Short Physical Performance Battery (continuous version), 0–3	2.03 (0.49), *n* = 168	2.16 (0.56), *n* = 140	1.91 (0.57), *n* = 168	2.00 (0.6), *n* = 133	^b^0.08 (− 0.01 to 0.17), *p* = 0.08, *n* = 273
Self-reported activity ease ^a^Activity Measure Post Acute Care, sum of basic mobility and daily activity components, 0–200	118.2 (18.2), *n* = 167	118.5 (18.2) *n* = 137	116.0 (16.5), *n* = 167	116.2 (19.9), *n* = 134	^b^0.18 (−2.89 to 3.26), *p* = 0.91, *n* = 270
Self-reported participation ^a^Late Life Disability Instrument, performance plus limitation scaled scores, 0–200	114.1 (20.7), *n* = 164	121.0 (24.5), *n* = 144	107.0 (24.5), *n* = 161	114.4 (25.6), *n* = 136	^b^1.37 (− 2.56 to 5.32), *p* = 0.49, *n* = 273

^a^Higher scores reflect better performance; ^b^Between-group difference in units of the outcome from linear regression models adjusted for baseline values for that outcome

**Table 3:** Baseline and 12-Month Scores on Secondary Outcomes

	Intervention (*n* = 168), mean (SD), *n* or *n* (%)		Control (*n* = 168), mean (SD), *n* or *n* (%)		12-month between-group difference adjusted for baseline (95% CI), *p*, *n*
	Baseline *n* = 168	12 month *n* = 146	Baseline *n* = 168	12 month *n* = 138	
Balance and mobility: performance					
^a^Short physical performance battery, 0–12, mean (SD)	7.9 (2.8) *n* = 168	8.9 (3.1) *n* = 140	7.4 (3.1) *n* = 168	8.0 (3.2) *n* = 133	^c^*0.70 (0.17 to 1.22), p = 0.009*, *n* = 273
^b^Sit to stand, time for 5 sit to stands, sec, mean (SD)	20.5 (10.0) *n* = 168	17.1 (10.3) *n* = 141	22.8 (11.0) *n* = 168	20.6 (11.3) *n* = 136	^c^**−** *2.6 (− 4.7 to − 0.5), p = 0.016*, *n* = 276
^b^Gait speed, time to walk 4 m, sec, mean (SD)	5.8 (2.5) *n* = 168	5.3 (3.6) *n* = 141	6.7 (4.1) *n* = 168	6.0 (4.0) *n* = 134	^c^− 0.07 (− 0.69 to 0.56), *p* = 0.832, *n* = 275
^a^Standing balance, sum of feet together, semi-tandem, tandem stance times, sec, 0–30, mean (SD)	25.5 (6.2) *n* = 168	25.5 (7.8) *n* = 142	24.9 (6.6) *n* = 168	24.7 (7.3) *n* = 135	^c^0.51 (− 0.86 to 1.87), *p* = 0.47, *n* = 277
^a^Single leg stance time, sec, 0–10, mean (SD)	3.8 (4.4) *n* = 168	4.9 (4.5) *n* = 141	3.5 (4.4) *n* = 168	3.7 (4.2) *n* = 135	^c^*0.89 (0.09 to 1.70), p = 0.03*, *n* = 276
^a^Maximum balance range, mm, mean (SD)	136.2 (54.5) *n* = 166	145.9 (60.5) *n* = 141	131.1 (55.6) *n* = 163	133.8 (62.4) *n* = 135	^c^6.3 (−5.3 to 17.8), *p* = 0.286 *n* = 271
^b^Coordinated stability, total distance of inside corners missed, mm, mean (SD)	85.4 (98.9), *n* = 162	52.9 (80.3), *n* = 140	79.0 (90.0), *n* = 158	64.9 (80.7), *n* = 134	^c^− 12.30 (− 29.67 to 5.07), *p* = 0.16, *n* = 262
^a^Step test, number of steps onto 7.5 cm block 15 s, average both legs, mean (SD)	9.8 (2.6) *n* = 168	9.0 (3.0) *n* = 142	10.7 (4.0) *n* = 168	9.6 (3.1) *n* = 134	^c^− 0.16 (− 0.75 to 0.44), *p* = 0.609, *n* = 76
^b^Choice stepping reaction time, time to complete routine, sec, mean (SD)	44.9 (19.7) *n* = 166	42.7 (19.5) *n* = 141	47.1 (19.7) *n* = 168	49.1 (20.0) *n* = 133	^c^*− 4.66 (− 7.5 to − 1.8), p = 0.001 n* = 272
^a^No walking aid used during assessment, *n* (%)	134 (80%) *n* = 168	116 (82%) *n* = 141	129 (77%) *n* = 168	107 (80%) *n* = 134	^e^1.46, 0.70 to 3.03, *p* = 0.31, *n* = 275
Balance and mobility: reported activity ease					
^a^Activity measure post acute care basic mobility score, 0–100, mean (SD) follow-up at 3, 6, 9, and 12 months	60.2 (6.9), *n* = 167	60.8 (7.6), *n* = 148 61.3 (8.06) *n* = 140 61.0 (8.3) *n* = 139 60.4 (7.8) *n* = 137	58.7 (6.4), *n* = 167	59.7 (7.3), *n* = 151 59.3 (8.7) *n* = 141 59.9 (8.3) *n* = 138 59.2 (8.2) *n* = 136	^c^− 0.07, − 1.1 to 0.96, *p* = 0.90, *n* = 299
					^c^0.87, − 0.46 to 2.22, *p* = 0.20, *n* = 280
					^c^0.09, − 1.27 to 1.46, *p* = 0.90, *n* = 276
					^c^0.03, − 1.24 to 1.31, *p* = 0.96, *n* = 272
^a^Activity measure post acute care daily activity score, 0–100, mean (SD) follow-up at 3, 6, 9, and 12 months	58.1 (12.9), *n* = 167	59.5 (13.5), *n* = 148 60.5 (14.4), *n* = 139 58.2 (13.0), *n* = 139 58.0 (12.4), *n* = 137	57.3 (11.5), *n* = 167	57.2 (13.7), *n* = 149 57.4 (12.9), *n* = 136 56.3 (11.2), *n* = 138 56.8 (13.3), *n* = 134	^c^1.28, − 0.77 to 3.35, *p* = 0.22, *n* = 297
					^c^*2.13, 0.35 to 4.23, p = 0.046*, *n* = 274
					^c^1.15, − 0.95 to 3.25, *p* = 0.28, *n* = 276
					^c^0.21, − 2.05 to 2.48, *p* = 0.85, *n* = 270
^a^Activity measure post acute care basic mobility + daily activity, 0–200, mean (SD) follow-up at 3, 6, and 9 months	118.2 (18.2) *n* = 167	120.4 (19.4), *n* = 148 121.9 (20.8) *n* = 139 119.2 (19.7) *n* = 139	116.0 (16.5) *n* = 167	117.0 (19.3) *n* = 149 116.9 (19.9) *n* = 136 116.2 (18.1) *n* = 138	^c^1.16, − 1.51 to 3.84, *p* = 0.39, *n* = 297
					^c^0.94, − 0.04 to 5.91, *p* = 0.053, *n* = 274
					^c^1.21, − 1.82 to 4.23, *p* = 0.433, *n* = 276
^b^Balance and mobility: activity difficulty from calendars, 9–45, mean (SD) follow-up at 3, 6, 9, and 12 months	22.4 (8.5), *n* = 158	22.2 (8.4), *n* = 147 21.2 (8.5), *n* = 144 21.2 (8.7), *n* = 142 21.6 (9.1), *n* = 137	23.0 (8.1), *n* = 150	22.6 (8.3), *n* = 143 23.1 (9.0), *n* = 147 23.5 (9.4), *n* = 139 23.7 (8.9), *n* = 136	^c^− 0.14, − 1.3 to 1.1, *p* = 0.82, *n* = 277
					^c^− 1.2, − 2.5 to 0.1, *p* = 0.07, *n* = 280
					^c^− *1.6, − 3.0 to 0.1, p = 0.04*, *n* = 271
					^c^− 1.3, − 2.8 to 0.2, *p* = 0.08, *n* = 262
Balance and mobility: self-reported participation					
^a^Late Life Disability Instrument, limitation scale, 0–100, mean (SD)	61.75 (13.17), *n* = 164	64.60 (14.9), *n* = 144	58.0 (15.5), *n* = 161	61.1 (15.7), *n* = 136	^c^1.42, − 1.3 to 4.2, *p* = 0.30, *n* = 273
^a^Late Life Disability Instrument, activity frequency scale, 0–100, mean (SD)	52.3 (9.0), *n* = 164	56.3 (11.1), *n* = 145	49.2 (10.3). *n* = 161	53.4 (11.5), *n* = 137	^c^0.15, − 1.57 to 1.86, *p* = 0.87, *n* = 275
Fall risk					
^b^Physiological profile assessment, total score, mean (SD)	0.37 (1.19) n = 168	0.55 (1.14) n = 146	0.56 (1.33) n = 168	1.06 (1.40) *n* = 136	^c^− *0.38*, − 0.61 to − 0.14, *p* = 0.002, *n* = 282
^a^Visual contrast sensitivity, score, mean (SD)	19.76 (2.39) *n* = 167	20.21 (2.43) *n* = 141	19.51 (2.45) *n* = 168	19.06 (3.75) *n* = 137	^c^*0.92, 0.26 to 1.58, p = 0.006*, *n* = 277
^b^Postural sway on foam total length, mm, mean (SD)	115.24 (59.58) *n* = 163	120.66 (54.34) *n* = 130	114.62 (64.28) *n* = 160	135.61 (90.79) *n* = 120	^c^− 0.15, − 31.8 to 0.72, *p* = 0.061, *n* = 248
^a^Knee extension strength, kg, mean (SD)	14.7 (6.0) *n* = 160	17.8 (8.01) *n* = 135	14.7 (6.1) *n* = 149	16.03 (7.4) *n* = 128	^c^*1.81 (0.32 to 3.310, p = 0.017*, *n* = 250
^b^Lower limb proprioception, degrees of error, mean (SD)	1.69 (1.24) *n* = 160	1.66 (1.06) *n* = 135	1.72 (2.09) *n* = 157	1.97 (1.46) *n* = 128	^c^**−** *0.33 (− 0.64 to − 0.10), p = 0.042*, *n* = 254
^b^Hand reaction time, sec, mean (SD)	254.44 (81.63) *n* = 168	261.48 (69.97) *n* = 141	274.03 (91.46) *n* = 168	279.51 (70.59) *n* = 134	^c^− 15.02 (− 30.61 to 0.58), *p* = 0.059, *n* = 275
Physical activity					
^a^Total habitual physical activity, h/week, mean (SD)	27.53 (15.95) *n* = 168	25.87 (17.71) *n* = 145	25.99 (17.39) *n* = 167	22.90 (16.58) *n* = 137	^c^2.09 (− 1.44 to 5.62), *p* = 0.245, *n* = 282
^a^Home exercise, h/week, mean (SD)	0.77 (2.14) *n* = 168	0.87 (1.94) *n* = 146	0.74 (1.14) *n* = 166	0.47 (1.14) *n* = 138	^c^*0.39 (0.02 to 0.77*), *p = 0.04*, *n* = 284
^a^Planned activities (including walking), h/week, mean (SD)	3.19 (3.61) *n* = 168	3.95 (5.77) *n* = 146	3.55 (5.7) *n* = 167	2.57 (3.12) *n* = 137	^c^*1.46 (0.42 to 2.50), p = 0.006*, *n* = 283
Pain					
^a^Little or no pain in fracture area, *n* (%)	71 (43%) *n* = 167	55 (38%) *n* = 146	75 (46%) *n* = 164	51 (37%) *n* = 137	^d^0.92 (0.59 to 1.43), *p* = 0.72, *n* = 282
Body mass index, kg/m^2^, mean (SD)	25.9 (5.4) *n* = 167	26.6 (5.6) *n* = 143	26.3 (5.6) *n* = 167	26.3 (6.2) *n* = 137	^c^0.60 (− 0.08 to 1.29), *p* = 0.085, *n* = 279
Fall-related self-efficacy					
^a^Self-rated balance excellent/ very good, *n* (%)	33 (20%) *n* = 168	29 (20%) *N* = 143	23 (14%) *n* = 165	23 (17%) *n* = 138	^d^1.18 (0.76 to 1.82), *p* = 0.46, *n* = 281
^a^Self-rated fear of falling not at all/a little, *n* (%)	90 (54%) *n* = 168	81 (57%) *n* = 143	79 (48%) *n* = 165	73 (53%) *n* = 138	^d^1.13 (0.73 to 1.75, *p* = 0.59, *n* = 281
^b^Concerns about falling, Falls Efficacy Scale Global, 7–28, mean (SD)	11.45 (4.46) *n* = 168	11.70 (4.61) *n* = 145	11.8 (4.17) *n* = 165	12.44 (4.92) *n* = 138	^c^− 0.36 (− 1.28 to 0.55), *p* = 0.435, *n* = 283
Mood					
^b^Geriatric Depression Scale, 0–5, mean (SD)	1.1 (1.3), *n* = 164	0.6 (1.1), *n* = 145	1.2 (1.3) *n* = 161	0.84 (1.1) *n* = 145	^c^− 0.18 (− 0.41 to 0.06), *p* = 0.14, *n* = 275
					^d^*0.58 (0.35 to 0.95), p = 0.03*, *n* = 275
^a^Positive and negative affect schedule, positive subscale, 10–50, mean (SD)	35.8 (7.4) *n* = 165	36.0 (7.6) *n* = 144	33.8 (8.4) *n* = 161	33.9 (7.8) *n* = 138	^c^0.98 (− 0.45 to 2.41), *p* = 0.18, *n* = 275
^b^Self-rated overall health at 12 months compared to baseline, *n* (%)		59 (40%) 65 (45%) 22 (15%) *n* = 146		33 (24%) 81 (59%) 23 (17%) n = 137	^d^*0.58 (0.37 to 0.91), p = 0.017*, *n* = 283
Better					
Much the same					
Worse					
Stage of motivational readiness for change					
^a^Physical activity, *n* (%)	*n* = 166	*n* = 145	*n* = 164	*n* = 138	^d^*1.66 (1.09 to 2.55), p = 0.019*, *n* = 283
Pre-contemplation	13 (8%)	21 (14%)	14 (9%)	39 (28%)	
Contemplation	76 (45%)	33 (23%)	74 (45%)	31 (22%)	
Preparation	19 (12%)	22 (15%)	32 (19%)	16 (12%)	
Action	18 (11%)	8 (6%)	11 (7%)	3 (2%)	
Maintenance	40 (24%)	61 (42%)	33 (20%)	49 (36%)	
Community outings					
^a^Outings per month to movies, etc., mean (SD)	2.3 (3.3)	2.7 (3.6)	2.5 (3.8)	1.8 (2.4)	^e^*1.47 (1.11 to 1.95), p = 0.007*, *n* = 284
^a^Visits to relatives per month, mean (SD)	6.1 (8.8) *n* = 167	3.9 (5.8) *n* = 146	5.1 (6.9) *n* = 165	2.7 (3.7) *n* = 138	^e^1.10 (− 0.004 to 2.19), *p* = 0.051, *n* = 284

^a^Higher scores reflect better performance; ^b^Lower scores reflect better performance; ^c^Between-group difference in units of the outcome from linear regression models adjusted for baseline values for that outcome; ^d^Between-group difference expressed as odds ratio from ordinal regression models adjusted for baseline values for that outcome; ^e^Between-group difference expressed as incidence rate ratio (IRR) from negative binomial regression comparing adjusted for baseline values for that outcome. Italic values indicate between-group differences that are statistically significant (*p* < 0.05)

### Intervention Received

Participants randomized to the intervention group received an average of 8.4 (SD 2.9, median 10, range 0 to 13) home visits and 4.3 (SD 1.9, median 5, range 0 to 10) phone calls from the study physiotherapists. Table 4 shows the nature of the exercises prescribed, dose agreed to by participants and dose completed for the intervention group at four time points. Supplementary Table 3 shows that the exercise intervention was well received by participants. Factors limiting exercise reported by study physiotherapists and participants are shown in Supplementary Table 4. Progress on the goals set by the intervention group is shown in Supplementary Table 5.

**Table 4:** Intervention Received by Intervention Group (*n* = 164), all data are mean (SD), Range, *n*

	Month 1	Month 3	Month 8	Month 11
Prescribed exercises				
Number of different exercises	6.4 (1.2), 2–10, *n* = 160	7.0 (1.4), 3–10, *n* = 133	6.9 (1.7), 2–10, *n* = 124	7.0 (1.7), 2–10, *n* = 110
Repetitions per session	91.5 (34.2), 5–188, *n* = 160	124.0 (56.0), 15–323, *n* = 133	124.8 (62.2), 27–318, *n* = 124	133.5 (63.0), 32–318, *n* = 110
Sessions per week agreed	4.5 (1.8), 2–14, *n* = 155	4.3 (1.8), 2–14, *n* = 128	4.0 (1.9), 1–14, *n* = 118	3.9 (1.6), 1–7, *n* = 110
Completed exercises				
Sessions per week undertaken	4.2 (2.1), 0–14, *n* = 155	3.9 (2.1), 0–14, *n* = 128	3.7 (2.1), 0–14, *n* = 118	3.5 (2.0), 0–7, *n* = 110
Percentage of prescribed exercises completed	84.3 (26.3), 0–100, *n* = 155	77.4 (27.2), 0–100, *n* = 128	72.2 (29.1), 0–100, *n* = 118	70.6 (30.4), 0–100, *n* = 110
Calculated weekly repetitions completed	380.1 (277.1), 0–1617, *n* = 155	467.3 (401.3), 0–2457, *n* = 128	401.3 (343.5), 0–1624, *n* = 118	407.6 (366.7), 0–1918, *n* = 110

Four people did not commence a home exercise program due to health problems (*n* = 2), refusal of exercises (*n* = 1), or inability to contact (*n* = 1). Participants who commenced the intervention (*n* = 164) received an average of 8.6 (SD 2.7, range 1 to 13) home visits and 4.5 (SD 1.8 range 0 to 10) phone calls during the 12-month intervention period. The level of challenge of exercises prescribed at baseline was rated on a 3-point scale by the study physiotherapist as 2.4 (SD 0.6) for strength, 1.6 (0.6) for balance, and 1.4 (0.5) for endurance (*n* = 158). Exercise prescription included additional weight for 33 (21%) participants with an average weight of 2.2 kg (SD 0.9)

Thirty-three (20%) of the 164 intervention group participants who commenced the intervention attended a *Stepping On* self-management group program. Seventy-nine (48%) participants did not wish to attend the program in addition to receiving the home-based intervention, 5 (3%) had already attended it, 16 (10%) people could not access an available program, and 31 (19%) participants were ineligible for the program primarily due to their use of a walking frame or a hearing impairment. One hundred and nineteen people received individualized fall prevention advice in their homes from the study physiotherapists.

### Primary Outcomes

For the primary outcome of mobility-related disability, the adjusted mean between-group difference at 12 months was 0.08 points on a 0–3 scale (95% CI − 0.01 to 0.17, *p* = 0.08, *n* = 273) for the SPPB (continuous version), 0.18 points on a 0–200 scale (95% CI − 2.89 to 3.26, *p* = 0.91, *n* = 270) for the AM-PAC, and 1.37 points on a 0–200 scale (95% CI − 2.56 to 5.32, *p* = 0.49, *n* = 273) for the LLDI. There were no statistically significant differences for these three variables. Data are shown in Table 2. During the 12-month study period, 142 people (42% of participants) experienced 260 falls (Table 5). There was no difference between the number of falls in the intervention group compared to the control group (IRR 0.96, 95% CI 0.69 to 1.34, *p* = 0.83, *n* = 336).

**Table 5:** Number (percentage) of Participants Falling and Total Number of Falls of Different Types in Control and Intervention Groups During 12-Month Follow-up

	Intervention group (*n* = 168)	Control group (*n* = 168)	Unadjusted IRR (95% CI), *n* = 336^a^	IRR adjusted for physical activity exposure (95% CI), *n* = 333^b^	IRR adjusted for planned physical activity exposure (95% CI), *n* = 333^c^
Primary outcome					
Number of falls, *n*	131	129	0.96 (0.69 to 1.34), *p* = 0.830		
Secondary outcomes/exploratory analyses					
Number of falls, *n*	131	129		0.71 (0.44 to 1.14), *p* = 0.155	0.54 (0.28 to 1.05), *p* = 0.068
People with falls, *n* (%)					
0	96 (57%)	98 (58%)			
1	38 (23%)	36 (21%)			
2	22 (13%)	20 (12%)			
3	4 (2%)	9 (5%)			
≥ 4	8 (5%)	5 (3%)			
1+ falls	72 (43%)	70 (42%)	1.03 (0.80 to 1.32), *p* = 0.825^d^		
Falls indoors, *n*	63	85	0.70 (0.45 to 1.07), *p* = 0.101	0.38 (0.20 to 0.72), *p* = 0.003	0.35 (0.15 to 0.80), *p* = 0.013
Falls outdoors, *n*	68	44	1.48 (0.94 to 2.35), *p* = 0.09	1.56 (0.91 to 2.68), *p* = 0.102	1.05 (0.43 to 2.55), *p* = 0.912
Falls with fractures, *n*	12	18	0.64 (0.30 to 1.35), *p* = 0.237	0.51 (0.20 to 1.27), *p* = 0.146	0.32 (0.07 to 1.45), *p* = 0.138
Falls requiring medical care, *n*	42	39	1.02 (0.61 to 1.68), *p* = 0.938	0.86 (0.44 to 1.69), *p* = 0.668	0.50 (0.18 to 1.40), *p* = 0.186
Falls requiring hospital admission, *n*	16	18	0.85 (0.43 to 1.68), *p* = 0.638	0.73 (0.33 to 1.63), *p* = 0.440	0.36 (0.09 to 1.41), *p* = 0.144
Follow-up, days, mean (SD)	347.2 (66.6)	332.7 (91.2)			

^a^Between-group difference from negative binomial regression models comparing rates between groups adjusted for exposure: days of follow-up; ^b^Between-group difference from negative binomial regression models comparing rates between groups adjusted for exposure: days of follow-up × self-reported hours of physical activity from Incidental and Planned Exercise Questionnaire^28^; ^c^Between-group difference from negative binomial regression models comparing rates between groups adjusted for exposure: days of follow-up × self-reported hours of planned physical activity from Incidental and Planned Exercise Questionnaire^28^; ^d^Between-group difference from Poisson regression models comparing proportions between groups

### Secondary Outcomes

Between-group differences were evident in several secondary outcome measures of balance and mobility (SPPB (12-point version), single leg stance time, choice stepping reaction time, AM-PAC daily activity score at 6 months, activity difficulty at 9 months), fall risk (Physiological Profile Assessment (PPA) score), physical activity (home exercise, planned activities), mood (Geriatric Depression Scale, 6-item version), self-rated health (better than baseline), stage of motivational readiness for change, and community outings (outings to movies, etc.). These data are shown in Table 3.

### Additional Analyses

The impact of the intervention on falls was larger after adjustment for exposure (defined as the hours per week of planned physical activity) and approached statistical significance (exploratory analysis; IRR 0.54, 95% CI 0.28 to 1.05, *p* = 0.068). Pre-planned sub-group analyses revealed a greater impact on SPPB (continuous version) in those who walked more quickly at baseline (p for interaction = 0.045). There was no evidence of a differential impact of the intervention with respect to baseline gait speed for the other primary outcomes or with respect to past falls or cognitive function for any of the primary outcomes (p for interaction > 0.05. data not shown).

### Adverse Events

Musculoskeletal problems that impaired daily activities for two or more days, or required medical attention, were reported by six participants in the intervention group: two developed Achilles tendon pain, one a skin tear from the leg colliding with a hard object while exercising, one developed pain after spine extension prior to an exercise session and was found to have a compression fracture, one developed a fracture complication, one an exacerbation of knee pain after a stepping exercise. No other adverse events were reported. Adverse events were not monitored in the control group.

## DISCUSSION

This large trial of the home-based RESTORE program did not detect between-group differences compared to usual care for the primary outcomes of mobility disability or fall frequency. The improvements in several secondary outcome measures relating to balance and mobility, daily task difficulty, fall risk (Physiological Profile Assessment tool), physical activity, mood, self-rated health and community outings in the intervention group, suggest the RESTORE intervention warrants further investigation but should be interpreted with caution due to the risk of spurious findings from multiple testing. The relatively strong acceptability of the intervention by participants and relatively high adherence levels and rarity of adverse events (i.e., just one compression fracture that occurred in the warm-up (stretching) phase of the intervention), suggest that it is possible for physiotherapists to teach people recovering from fall-related fractures to safely undertake a home exercise program. The high proportion of people reporting health problems interfered with their exercise indicates the complexity of home exercise prescription in this population.

It is unclear why there was not an impact of the intervention on the primary outcome measures. Possible explanations are the mixed population, the time after fracture, the home-based un-supervised nature of the intervention, the sub-optimal intervention adherence and the other health problems developed by many participants over the trial period. It is also possible that participation and falls outcomes need more targeted interventions. More work is needed to establish the most responsive measures of outcomes in older people after fall-related fracture. The possible impact on some measures of balance and mobility reinforces the findings of two previous studies^12, 17^ reporting benefits of home exercise after hip fracture. In all three studies, the exercises were primarily “functionally relevant,” that is the exercise was undertaken in weight-bearing positions rather than more traditional lying or seated exercises.

This study is not without limitations. In particular, the drop-out rate was sub-optimal and may reflect the complexity of running trials in this population. The findings of the secondary and exploratory analyses need to be interpreted with caution due to the risk of spurious findings with multiple testing. The variability in the sample may mean that the study lacked statistical power for some secondary outcomes. The study cannot tell us the impact of a more highly supervised intervention or the impact if all participants had received the *Stepping On* element of the intervention. The relatively low recruitment rate, which also applies to many other trials, may limit the generalizability of results. Strengths of the study include the attempt to minimize the risk of bias through assessor blinding, concealed allocation to groups, and intention-to-treat analysis.

The exploratory finding that the intervention was protective on the rate of falls if analyses were adjusted for exposure warrants application in future trials. It is common in other areas of injury prevention to adjust for exposure (e.g., driving and sports injuries) but such an adjustment is not usually undertaken in fall prevention trials. The different impact of the intervention on outdoor versus indoor falls also requires investigation in future trials. Planned sub-group analyses suggested that the intervention may have a greater impact in people who walked more quickly at baseline. This may suggest less potential for improvement or a need for a more intensive intervention for those people who walk more slowly.

In conclusion, although there was no significant impact on the primary outcomes we consider that this and similar interventions warrant further investigation given the complexity and variability of the study population, the increasing global problem of falls and fractures in older adults and the need for interventions that could be scaled up for broader implementation.

## Electronic Supplementary Material

ESM 1 (DOCX 281 kb)

## References

[1] Handoll HH, Sherrington C, Mak JC (2011). Interventions for improving mobility after hip fracture surgery in adults. Cochrane Database Syst Rev.

[2] Magaziner J, Hawkes W, Hebel JR (2000). Recovery from hip fracture in eight areas of function. J Gerontol A Biol Sci Med Sci.

[3] Parker M, Johansen A, Parker M, Johansen A (2006). Hip fracture. BMJ..

[4] Ensrud KE (2013). Epidemiology of fracture risk with advancing age. J Gerontol A Biol Sci Med Sci.

[5] Auais MA, Eilayyan O, Mayo NE (2012). Extended exercise rehabilitation after hip fracture improves patients' physical function: a systematic review and meta-analysis. Phys Ther.

[6] **Diong J, Allen N, Sherrington C.** Structured exercise improves mobility after hip fracture: a meta-analysis with meta-regression. Br J Sports Med*.* 2015. 10.1136/bjsports-2014-094465 26036676

[7] Crotty M, Unroe K, Cameron ID, Miller M, Ramirez G, Couzner L (2010). Rehabilitation interventions for improving physical and psychosocial functioning after hip fracture in older people. Cochrane Database Syst Rev.

[8] Sherrington C, Tiedemann A, Cameron I (2011). Physical exercise after hip fracture: an evidence overview. Eur J Phys Rehabil Med.

[9] Hauer K, Specht N, Schuler M, Bartsch P, Oster P (2002). Intensive physical training in geriatric patients after severe falls and hip surgery. Age Ageing.

[10] Binder EF, Brown M, Sinacore DR, Steger-May K, Yarasheski KE, Schechtman KB (2004). Effects of extended outpatient rehabilitation after hip fracture: a randomized controlled trial. JAMA..

[11] Orwig DL, Hochberg M, Yu-Yahiro J (2011). Delivery and outcomes of a yearlong home exercise program after hip fracture: a randomized controlled trial. Arch Intern Med.

[12] Magaziner J, Mangione KK, Orwig D (2019). Effect of a Multicomponent Home-Based Physical Therapy Intervention on Ambulation After Hip Fracture in Older Adults: The CAP Randomized Clinical Trial. JAMA..

[13] Latham NK, Harris BA, Bean JF (2014). Effect of a home-based exercise program on functional recovery following rehabilitation after hip fracture: a randomized clinical trial. JAMA..

[14] Sherrington C, Lord SR, Herbert RD (2004). A randomized controlled trial of weight-bearing versus non-weight-bearing exercise for improving physical ability after usual care for hip fracture. Arch Phys Med Rehabil.

[15] Sherrington C, Lord SR (1997). Home exercise to improve strength and walking velocity after hip fracture: a randomized controlled trial. Arch Phys Med Rehabil.

[16] Cummings SM, Cooper R, Cassie K (2009). Motivational interviewing to affect behavioral change in older adults. Res Soc Work Pract.

[17] O’Halloran PDBF, Shields N, Wintle E, Taylor NF (2016). Motivational interviewing increases physical activity and self-efficacy in people living in the community after hip fracture: a randomized controlled trial. Clin Rehabil.

[18] Bischoff-Ferrari HA, Dawson-Hughes B, Platz A (2010). Effect of high-dosage cholecalciferol and extended physiotherapy on complications after hip fracture: a randomized controlled trial. Arch Intern Med.

[19] Sherrington C, Lord SR, Vogler CM (2014). A post-hospital home exercise program improved mobility but increased falls in older people: a randomised controlled trial. PLoS One.

[20] Sherrington C, Tiedemann A, Fairhall N, Close JC, Lord SR (2011). Exercise to prevent falls in older adults: an updated meta-analysis and best practice recommendations. NSW Public Health Bull.

[21] Sherrington C, Fairhall NJ, Wallbank GK (2019). Exercise for preventing falls in older people living in the community. Cochrane Database Syst Rev.

[22] Clemson L, Cumming RG, Kendig H, Swann M, Heard R, Taylor K (2004). The effectiveness of a community-based program for reducing the incidence of falls in the elderly: a randomized trial. J Am Geriatr Soc.

[23] Sherrington C, Fairhall N, Kirkham C (2016). Exercise and fall prevention self-management to reduce mobility-related disability and falls after fall-related lower limb fracture in older people: protocol for the RESTORE (Recovery Exercises and STepping On afteR fracturE) randomised controlled trial. BMC Geriatr.

[24] Prochaska JO, Redding CA, Evers KA, Glanz E, Rimer BK, Lewis FM (2002). The transtheoritical model and stage of change. Health behavior and health education: Theory, research, and practice.

[25] Onder G, Penninx BW, Ferrucci L, Fried LP, Guralnik JM, Pahor M (2005). Measures of physical performance and risk for progressive and catastrophic disability: results from the Women's Health and Aging Study. J Gerontol A-Biol Sci Med Sci.

[26] Gibson MJ, Andres RO, Isaacs B, Radebaugh T, Worm-Petersen J (1987). The prevention of falls in later life. Dan Med Bull.

[27] Bath PM, Lees KR, Schellinger PD (2012). Statistical analysis of the primary outcome in acute stroke trials. Stroke.

[28] Delbaere K, Hauer K, Lord S (2010). Evaluation of the Incidental and Planned Exercise Questionnaire (IPEQ) for older people. Br J Sports Med.

[29] Marcus BH, Selby VC, Niaura RS, Rossi JS (1992). Self-efficacy and the stages of exercise behavior change. Res Q Exerc Sport.

