# Supplementary material for: Exercise to Reduce Mobility Disability and Prevent Falls After Fall-Related Leg or Pelvic Fracture: RESTORE Randomized Controlled Trial
Source: J Gen Intern Med. 2020 Feb 3;35(10):2907–16. doi: 10.1007/s11606-020-05666-9 (PMC7573017; doi:10.1007/s11606-020-05666-9)

**Supplementary Material.**

**Supplementary Table 1. Intervention description using the Template for Intervention Description and Replication (TIDieR) checklist**

| **TIDieR Domain** | **Description** |
| --- | --- |
| **Brief name** | RESTORE: Recovery Exercises and STepping On afteR fracture intervention program |
| **Why** | This home-based exercise and fall-prevention education intervention used a self-management approach and aimed to reduce mobility-related disability and falls in older people following fall-related lower limb or pelvic fracture. The majority of exercise was undertaken independently at home, aiming for a balance between sufficient intensity for effectiveness and reduced cost for feasibility of implementation. The self-management approach aims to enhance uptake of the exercise program using goal-setting when desired by the participant and the education aspect involves *Stepping On* program attendance or individualized information focusing on safe community mobility and other risk factors for falls. |
| **What** | The exercises were primarily conducted while standing and were based on the Weight-bearing Exercise for Better Balance program, which is available at [www.webb.org.au](http://www.webb.org.au). For participants who were interested in setting mobility goals these were used to guide the choice and dose of exercises. The Physical Activity Stage of Change model was used by the study physiotherapists to guide their approach to encouraging ongoing home-based exercise participation. Exercises that primarily targeted postural control (balance) included standing with a narrower base (aiming for tandem or single leg stance), forwards and sideways stepping/walking, and graded reaching activities in standing. The lower limb extensor muscle groups (i.e., hip and knee extensors and ankle plantarflexors), which act to prevent collapse of the lower limb, were targeted with exercises aiming to enhance muscle strength and control. Strengthening exercises included sit-to-stand, forward and lateral step-ups onto a small block, and heel raises in standing. Where appropriate, resistance for strengthening exercises was added by the use of weight-belts worn around the waist or weighted vests. The use of upper limbs to support the body while exercising was minimized but was used where necessary to ensure safety. Participants were instructed on how to perform exercises with stable supports (such as a table) located nearby to hold onto if needed and, where appropriate, family members and/or carers were encouraged to assist with supervision of the exercise program. Participants were provided with a booklet of safety precautions, instructions and photographs of exercises for use in exercise sessions at home.  The education intervention was based on the *Stepping On* program manual [Stepping On: Building confidence and reducing falls, A community-based program for older people 2nd Edition](http://purl.library.usyd.edu.au/sup/9781920898755) (Clemson and Swann, Sydney University Press, 2008) and the book [Staying Power: Tips and Tools to Keep You on Your Feet](https://sup-estore.sydney.edu.au/jspcart/cart/Product.jsp?nID=489&nCategoryID=SR&searchTitle=&searchAuthor=clemson&searchCategory=null&searchISBN=null) (Clemson and Swann, Limelight Press, 2006) Participants were provided with fall prevention materials from *Stepping On* and if they wished a copy of Staying Power: Tips and Tools to Keep You on Your Feet. |
| **Who provided** | Two experienced physiotherapists delivered the exercise component of the RESTORE intervention. Where possible, participants attended the group-based *Stepping On* program delivered by the NSW Health Department. If not possible, individual fall prevention education was delivered by the study physiotherapists. |
| **How** | The physiotherapists visited participants to prescribe and modify a home exercise program up to ten times in the 12-month study period, with more frequent visits at the commencement of the program to ensure safety and enable tailoring of the program. Participants were also telephoned five times to ask about exercise program participation and address any issues that had arisen. |
| **Where** | Participants homes/ group-based *Stepping On* program in community venues for some participants |
| **When and how much** | Participants were asked to undertake a 20 to 30 minute program of lower limb balance and strengthening exercises at least three times per week at home for 12 months. The exact dose of exercise was negotiated with the participant at each review visit with the aim of progression. |
| **Tailoring** | The physiotherapist prescribed the level of difficulty and number of repetitions for each exercise after an assessment of the participant’s abilities. |
| **Modifications** | If a participant became unwell or had another admission to hospital, the program was resumed when the participant and the relevant professionals deemed him/her well enough to participate again. The optimal intensity and type of exercises for each individual participant was regularly assessed and adjusted by the study physiotherapists to ensure that the intervention remained challenging as the participant’s performance improved. |
| **How well** | Participants were provided with a logbook for recording exercises completed and effects of exercise (e.g., muscle soreness). |

**Supplementary Table 2. Secondary outcome measures.**

| **Outcome** | **Description** |
| --- | --- |
| **Balance and mobility: performance** | The 12-point version of the SPPB and the time to undertake each of the included tasks as well as the time the participant could stand unsupported in single leg stance was assessed. The Maximal Balance Range test assessed participants' ability to lean as far forward and backwards as possible. The Coordinated Stability test required participants to adjust the body position in a steady and coordinated manner when near the limits of their base of support. The Step Test required the participant to step onto and off a 7.5 cm block as many times as possible in 15 seconds. Choice stepping reaction time has been found to be a composite measure of risk of falling when assessed with an electronic device. This was measured in a modified manner with a portable mat. A mat with four white squares was placed in front of the standing subjects, and they were asked to make a standard number of steps with either foot to a particular square and back again, using a standardized script. The time for the entire routine was recorded in seconds. Walking aid use during the assessment was also recorded. |
| **Balance and mobility: self-reported activity ease** | ^a^AM-PAC basic mobility score and daily activity score were assessed separately at 3, 6, 9, 12 months and the combined score was examined at 3, 6, 9 months. Self-reported activity difficulty was also reported from the monthly calendars in which participants were asked to rate level of difficulty undertaking 9 daily tasks on a 5-point scale. |
| **Balance and mobility: self-reported participation** | ^a^Late Life Functioning and Disability Instrument, Disability Component limitation and activity frequency scales were examined separately at 12 months. |
| **Falls** | Falls were further assessed by comparing the proportion of people experiencing one or more falls over the 12-month follow-up period in the intervention and control groups. Consequences of falls were assessed by the rate of fractures, falls requiring medical care and falls requiring hospitalization. |
| **Fall risk** | Risk of falling was assessed using the Physiological Profile Assessment, which includes measures of knee extension strength, postural sway, lower limb proprioception, reaction time and visual contrast sensitivity, and its components. |
| **Physical activity levels** | The Incidental Physical Exercise Questionnaire was used and the hours per week of total habitual physical activity, home exercise and planned activity were reported. |
| **Pain** | Participants were asked to rate the extent of pain in the fracture area on a 5-point scale. |
| **Body mass index** | Body mass index (BMI) was calculated on the basis of measured or reported weight and height as both high and low BMI are problematic for older adults. |
| **Fall-related self-efficacy** | Self-rated balance and fear of falling were assessed on a five-point scale. Level of concern about falling when carrying out a range of activities was rated on a 4-point scale using the Falls Efficacy Scale-Global |
| **Mood** | Mood was assessed using the Geriatric Depression Scale (6-item version) and Positive and Negative Affect Schedule. |
| **Self-rated overall health** | Participants were asked to rate their overall health compared to 12 months earlier. |
| **Readiness for change** | The stage of motivational readiness for change was assessed with the *Physical Activity Stages of Change Questionnaire.*^27^ |
| **Community outings** | Outings per month to movies or similar and to visit relatives was assessed by questionnaire. |

**Supplementary Table 3: Participant impressions of the intervention**

| Question | Response on 5 point scale strongly disagree (1) to strongly agree (5), mean (SD), range, n |
| --- | --- |
| I enjoyed the exercise program | 4.2 (0.8), 1-5, n=112 |
| I found the exercises easy to do | 3.8 (0.9), 1-5, n=112 |
| I feel the exercises are helpful in preventing falls | 4.2 (0.7), 1-5, n=112 |
| I feel the exercises are helpful in improving or maintaining my everyday mobility | 3.6 (0.9), 1-5, n=112 |
| I enjoy exercising | 3.9 (0.9), 2-5, n=112 |
| The exercise program was easily included in my weekly routine | 3.9 (0.9), 1-5, n=112 |
| The equipment provided was easy to use | 3.9 (0.9), 1-5, n=107 |
| I completed all of the prescribed exercises | 3.4 (1.3), 1-5, n=112 |
| I am confident that I can continue to exercise at least 3 times per week | 3.9 (1.0), 0-5, n=111 |

**Supplementary Table 4: Factors limited exercise prescription (n=112), multiple options allowed**

| **Factor** | **Reported by physiotherapist, n (%)** | **Reported by participant, n (%)** |
| --- | --- | --- |
| Illness or injury |  | 90 (80%) |
| Hospital admission | 36 (32%) |  |
| Medical issues | 68 (61%) |  |
| Medical condition worsened by exercise |  | 32 (29%) |
| Pain:  back pain  knee pain  other pain | 40 (36%)  42 (38%)  56 (50%) |  |
| Impaired cognition | 10 (9%) |  |
| Mental health | 19 (17%) |  |
| Bereavement | 17 (15%) |  |
| Moved house | 13 (12%) |  |
| Travel | 29 (26%) | 11 (10%) |
| Carer roles | 15 (13%) |  |
| Not interested | 19 (17%) |  |
| Falls | 15 (13%) |  |
| Doctor’s advice |  | 7 (6%) |
| Too busy |  | 23 (21%) |
| Too tired |  | 21 (19%) |
| Exercises too hard |  | 2 (2%) |
| Exercises too easy |  | 1 (1%) |
| Exercises not needed |  | 7 (6%) |
| Couldn’t see benefit |  | 10 (9%) |
| Don’t like exercise |  | 2 (2%) |
| Don’t like exercise alone at home |  | 14 (13%) |
| Forgot |  | 3 (3%) |

**Supplementary Table 5: Goals set and achieved^a^, all data are mean (SD), range, n**

|  | **Baseline** | **Follow up 1** | **Follow up 2** |
| --- | --- | --- | --- |
| **Goal 1** |  |  |  |
| importance | 8.3 (1.8), 3-10, n=66 | 8.5 (2.1), 0-10, n=47 | 9.1 (1.0), 8-10, n=10 |
| performance | 3.1 (2.1), 0-8, n=66 | 6.1 (2.8), 0-10, n=47 | 7.7 (2.1), 3-10, n=10 |
| satisfaction | 2.5 (2.1), 0-7, n=66 | 2.5 (2.1), 0-7, n=47 | 8 (2.2), 3-7, n=10 |
| **Goal 2** |  |  |  |
| importance | 8.1 (2.2), 2-10, n=41 | 8.1 (2.6), 1-10, n=32 | 9 (1.1), 8-10, n=6 |
| performance | 3.2 (2.2), 0-10, n=41 | 5.1 (3.2), 0-9, n=32 | 8.5 (0.7), 8-9.5, n=6 |
| satisfaction | 2.1 (2.1), 0-8, n=41 | 5.2 (3.3), 0-10, n=31 | 9.3 (0.8), 8-10, n=6 |

^a^Participant-reported importance, performance and satisfaction with each of two goals reported on a 10-point scale. Most goals set related to walking to a different location and with more confidence.

**Supplementary Figure 1: Study recruitment methods**


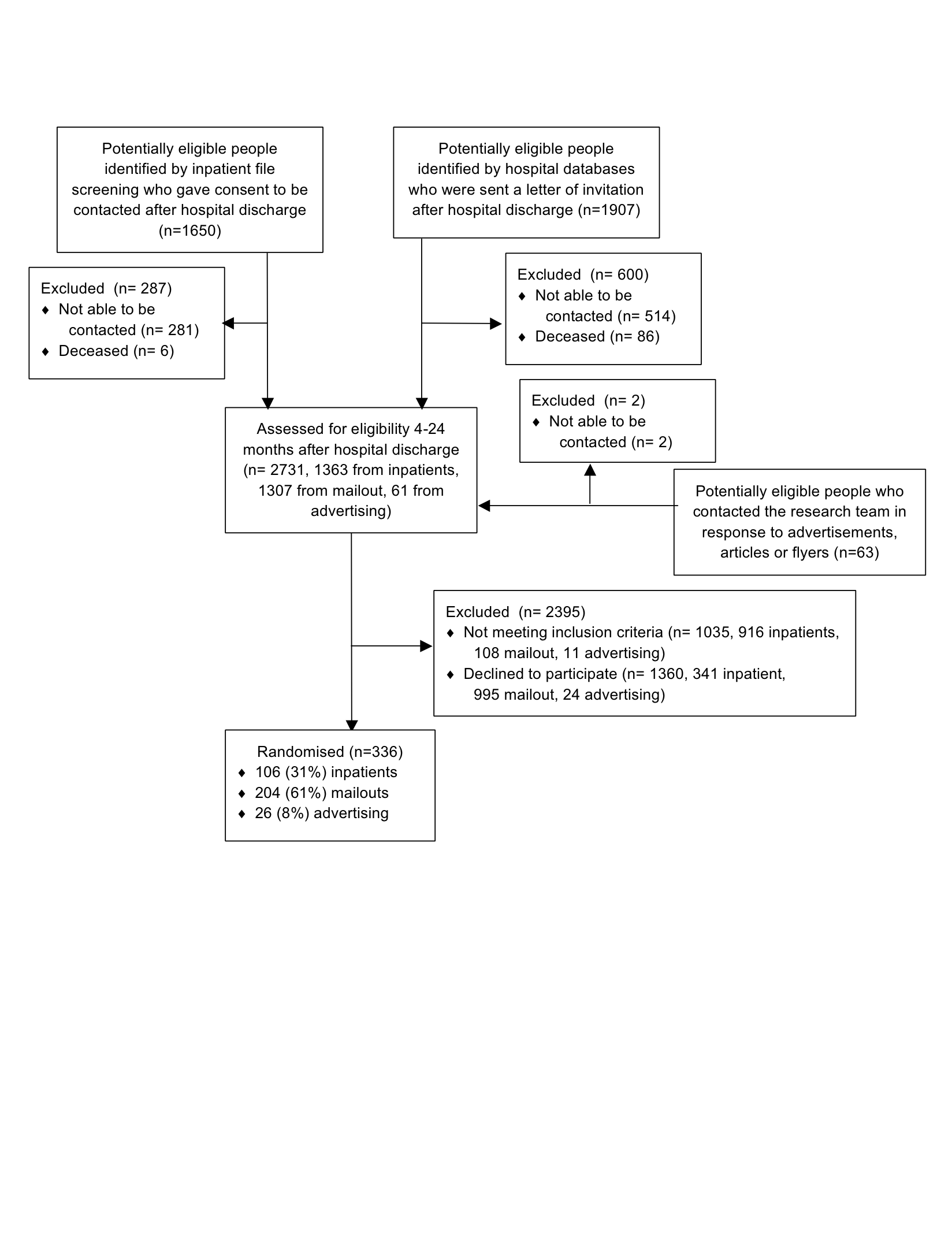

Supplement: Supplementary file 1 — (DOCX 281 kb) [file 11606_2020_5666_MOESM1_ESM.docx]
